# 

**Published:** 1882-01

**Authors:** 


SOCIETY MEETINGS.

Chicago Medical Society—Mondays, Jan. 2 and 16.

West Chicago Medical Society—Mondays, Jan. 9 and 23.

Biological Society—Wednesday, Jan. 4.

CLINICS.

MONDAY.

Eye and Ear Infirmary—2 p. m., Ophthalmological, by Prof. Holmes; 3 p. m., Otological, by Prof. Jones.

Mercy Hospital—2 p. m., Medical, Profs. Hollister and Quine.

Woman's Medical College—2 p. m., Dermatological and Venereal, by Prof. Maynard; 3 p. m., Diseases of the Chest, Prof. Ingals.

TUESDAY.

Rush Medical College—3 p. m., Dermatological and Venereal, by Prof. Hyde.

Cook County Hospital—2 to 4 p. m., Medical and Surgical Clinics.

Mercy Hospital—2 p. m., Surgical Clinic, by Prof. Andrews.

WEDNESDAY.

Chicago Medical College—2 p. m., Eye and Ear, by Prof. Jones.

Rush Medical College—2 p. m., Medical, by Dr. Bridge; 3 p. m., Ophthalmological and Otological, by Prof. Holmes; 3:30 to 4:30 p. m., Diseases of the Chest, by Dr. E. Fletcher Ingals.

THURSDAY.

Chicago Medical College—2 p. m., Gynæcological, Prof. Jenks.

Rush Medical College—2 p. m., Diseases of Children, by Dr. Knox; 3 p. m., Diseases of the Nervous System, by Prof. Lyman.

Eye and Ear Infirmary—2 p. m., Ophthalmological, by Dr. Hotz.

Woman's Medical College—3 p. m., Surgical, by Prof. Owens.

FRIDAY.

Cook County Hospital—2 to 4 p. m., Medical and Surgical Clinics.

Mercy Hospital—2 p. m., Medical, by Prof. Davis.

SATURDAY.

Rush Medical College—2 p. m., Surgical, by Prof. Gunn; 3 p. m., Orthopœdic, by Prof. Owens.

Mercy Hospital—2 p. m., Surgical Clinic, by Prof. Andrews.

Chicago Medical College—3 p. m., Neurological, Prof. Jewell.

Woman's Medical College—11 a. m., Ophthalmological, by Prof. Montgomery; 2 p. m., Gynæcological, by Prof. Fitch.

Daily Clinics, from 2 to 4 p. m., at the Central Free Dispensary, and at the South Side Dispensary.

